# Anti-diabetic and anti-oxidant potential of aged garlic extract (AGE) in streptozotocin-induced diabetic rats

**DOI:** 10.1186/s12906-016-0992-5

**Published:** 2016-01-19

**Authors:** Martha Thomson, Khaled K. Al-Qattan, Divya JS, Muslim Ali

**Affiliations:** Department of Biological Sciences, Faculty of Science, Kuwait University, P.O. Box 5969, 13060 Safat, Kuwait

**Keywords:** Aged garlic extract, Diabetes, Anti-diabetes

## Abstract

**Background:**

Although aged garlic extract (AGE) shares some active components with fresh garlic and in spite of its palatability and milder side effects, the anti-diabetic and related anti-oxidant properties of AGE have not been investigated extensively, and the reported findings are inconsistent.

This study investigated the anti-diabetic effects of 3 incremental doses of AGE in streptozotocin (STZ)-induced diabetic rats (fasting blood sugar > 20 mM).

**Method:**

Diabetic rats were divided into a control diabetic group (CD) and AGE-treated diabetic group (AGE-D). The AGE-D was divided into 3 groups and accordingly treated with AGE i.p. at 100, 300 and 600 mg/kg daily for 8 weeks. A control normal group (CN) was also included for reference.

**Results:**

Compared to the CN group, the CD group showed significant loss of body weight (over 50 %); and decreased serum insulin concentration (10 fold) and total anti-oxidant level and catalase activity (45–70 %) in serum, kidney and liver. Conversely, the CD rats had an elevated blood glucose (nearly 4 fold), serum cholesterol (nearly 2 fold) and triglycerides (>2 fold), erythrocyte glycated hemoglobin (GHb, 3 fold) and kidney and liver lipid peroxidation (MDA levels). Treatment with AGE positively reversed the diabetic changes in the targeted parameters to levels significantly lower than those measured in the CD group and the degrees of attenuation were almost dose dependent especially with the two higher doses.

**Conclusion:**

AGE exhibits a dose-dependent ameliorative action on indicators of diabetes in STZ-induced diabetic rats.

## Background

Fresh garlic (*Allium sativum L.*), a widely used condiment and spice, is well known for its health-promoting properties as reported in numerous human and animal studies [1–14]. Garlic’s health-promoting properties, which are of interest and fall within its antidiabetic potentials, are: hyperinsulinemia, hypoglycemia, hypocholesterolemia, hypotriglyceridemia and anti-glycation and anti-lipid peroxidation actions, in addition to promoting total anti-oxidant level and catalase activity. In spite of having these valuable ameliorative actions, fresh garlic consumption is, unfortunately, flawed by the manifestation of unpleasant side effects which significantly deter from its intake as a medicinal supplement. Amongst others, these include unpleasant and irritable gastrointestinal symptoms plus strong repellent breath and body odor; side effects which result from garlic oil soluble sulfur-containing compounds. Another deterrent to the use of fresh garlic is its form and preparation, in addition to the inconsistency in the dose of ingested active ingredients.

Aged garlic extract (AGE), produced by storing sliced and macerated fresh garlic at room temperature for 20 months, is odorless and upon ingestion causes minimal gastrointestinal irritability and socially unacceptable properties. These preferable attributes of AGE seem to result from the aging process which reduces the oil-soluble odorous sulfur compounds and enhances the content of water-soluble compounds [15]. AGE is also available over the counter in a form suitable and ready for intake with a known dose concentration. What is also interesting about AGE is that it contains many of the bioactive compounds found in fresh garlic including the water-soluble allyl amino acid derivatives, stable lipid-soluble allyl sulfides, flavonoids and saponins, and most importantly and to a large extent, S-allylcysteine (SAC) and S-allylmercaptocysteine (SAMC) [15, 16].

In vitro and in vivo studies on AGE have demonstrated potent anti-thrombotic [17] and anti-oxidant [18] activities. In relation to the anti-thrombotic activity, AGE and its constituents have been shown to inhibit ADP-induced platelet aggregation in a dose dependent manner [19]. Inhibition of platelet aggregation by AGE has been reported to involve multiple mechanisms including changes in intra-platelet [Ca^+2^], increase in intracellular cAMP and reduction in the interaction of fibrinogen with the CPIIb/IIIa receptor [19, 20]. In relation to anti-oxidant properties, several studies have demonstrated that AGE and its components have potent anti-oxidant activity [14]. This activity is suggested to be the result of scavenging free radicals and enhancing the cellular anti-oxidant systems [18–21]. Furthermore, in animal models, AGE has been shown to lower oxidative stress (OS) by increasing endogenous anti-oxidant enzymes, such as catalase and glutathione peroxidase (GPx), [22] and lowering pro-oxidant enzymes such as xanthine oxidase and NADPH oxidase [23]. AGE can also reduce the formation of superoxide and lipid peroxidation and thus alleviate OS [24].

Diabetes mellitus (DM), an increasingly prevalent medical condition, is characterized by hyperglycemia, dyslipidemia and OS. Clinical and experimental studies have shown that diabetic hyperglycemia is a major source of OS, and that increased generation of free radicals plays a significant role in the pathogenesis and complications of DM [25–28]. Urate and vitamin C, major contributors to serum anti-oxidant activity, have shown to be significantly reduced in both insulin-dependent DM and non-insulin-dependent DM [29]. The basic mechanisms by which chronic hyperglycemia promote increased OS have been suggested to include glucose auto-oxidation, formation of advanced glycation end-products (AGEs) and increased activity of the sorbitol pathway [28–30].

During the last 20 years, many studies have investigated the anti-diabetic potential of fresh garlic. Our group and others have shown that raw garlic extract is active in reducing hyperglycemia and alleviating many diabetic indicators, such as dyslipidemia and OS in both animal models and humans [10, 13, 31–35]. However, few studies have examined the effects of AGE in diabetes and those reports have described variable effects of AGE on the classical indicators of DM. For example, Shiju and coworkers [36] reported that treating streptozotocin (STZ)-induced diabetic rats with 500 mg/kg of AGE for 12 weeks resulted in no change in blood glucose although hemoglobin glycation (GHb) was clearly reduced. In contrast, Balamash et al. [37] observed that treating type 2 DM patients with 3000 mg of AGE daily for 3 months did not affect blood glucose, GHb or the serum lipid profile in these patients, although lipid peroxidation, an indicator of OS, was reduced.

Based on its acceptable palatability and milder side effects, content of potent bioactive components and conflicting reported effects in DM, the present study was carried out to assess the anti-diabetic potential of AGE by conducting an 8-week daily intraperitoneal (IP) three incremental dose–response study in STZ-induced diabetic rats. The doses investigated were selected based on a pilot study. In addition to examining the effect of AGE on the general indicators of diabetes, such as serum insulin and glucose, erythrocyte GHb, food and water intake, urine output in addition to body weight changes, this study also looked at the indicators of OS and dyslipidemia to further delineate the scope of the anti-diabetic activity of this garlic preparation.

## Methods

### Chemicals and kits

AGE (Kyolic AGE) was purchased as a liquid suspension (Wakunaga, USA) containing 240 mg AGE/ml. All analysis kits were purchased from the different sources as indicated in their respective usage in this section.

### Treatment of rats

Healthy 3 month old male Sprague–Dawley rats (150–180 g BW) maintained on standard rodent diet and tap water *ad libitum* were used in the study. The rats were kept under standard ambient conditions (23 ± 2 °C, 12 h light, 12 h darkness).

Diabetes was induced in 60 rats by a single IP injection of STZ, 60 mg/kg in 0.5 ml of 0.01 M sodium citrate buffer, pH 4.5. After 5 days, blood glucose was quantitated after overnight fasting in a drop of tail-blood under mild diethyl ether sedation using One-Touch Ultra-Easy Glucometer (LifeScan, UK).

Diabetic rats (blood sugar level > 20 mM) were divided into four groups (each, *n* = 12–16): Control diabetic (CD) group injected IP daily with normal saline (NS); low-dose AGE-diabetic (AGE100-D) group injected IP daily with 100 mg AGE /kg; medium dose AGE-diabetic (AGE300-D) group injected IP daily with 300 mg AGE/kg; high dose AGE-diabetic (AGE600-D) group injected IP daily with 600 mg AGE/kg. A group of control normal rats (CN) that received NS was included as a reference.

The use of rats was in full compliance with the Guide for the Care and Use of Laboratory Animals [38] and all experimental protocols were approved by the Animal Care and Use Committee at Faculty of Science–Kuwait University.

### Measurements, tissue preparation and biochemical assays

The body weight of the rats in all groups was recorded before the start of the study and weekly during the experimental period. Twenty-four h water and food intake were measured weekly and 24 h urine output was measured in each treatment group at 8 weeks by housing the rats in metabolic cages.

After 8 weeks, the rats were sacrificed after overnight fast under Sodium Pentobarbitone (10 mg/kg, May & Baker, England). The blood was collected by cardiac puncture into two separate tubes for serum and red blood cell (RBC) preparation. For serum, 3 ml of blood was collected in a tube and allowed to clot for 30 min at room temperature. The tubes were centrifuged at 1000 × g for 15 min and the serum was separated, and stored as 0.5 ml aliquots in Eppendorf tubes at −80 °C for later analysis. For RBCs, 1 ml of whole blood was collected in a separate glass tube containing 0.2 ml of 3.8 % sodium citrate as an anticoagulant and mixed well. The tubes were centrifuged at 1000 × g for 10 min and the plasma and buffy coat were carefully removed from the cells. RBCs was mixed well and analyzed for GHb on the same day.

Immediately after sacrifice, the liver and kidneys of each rat were collected separately. The capsule of each kidney was removed and the tissues were washed with buffer, blotted with filter paper and stored at −40 °C for later analysis. One gram of kidney or liver tissue was diced and mixed with 3 ml Tris–HCl (0.05 M, pH = 7.6). The mixture was homogenized, allowed to stand on ice for a few minutes and then centrifuged for 15 min at 8000 × g at 4 °C. The supernatant was stored in small aliquots at −40 °C for later analysis.

Glucose levels in whole blood were determined after overnight fasting at 2, 4, 8 weeks as described above. Total water-soluble antioxidants were determined by the method of Rice-Evans and coworkers as modified by Drobiova *et al.* [31] based on inhibition of the oxidation of 2, 2 -azinobis-(3-ethylbenzothiazoline-6-sulfonic acid) (ABTS). Serum insulin was determined by ELISA using kits supplied by SPIbio (France). Total serum cholesterol and triglycerides were determined spectrophotometrically using test kits from Randox (USA). Serum GHb was determined by an affinity chromatography method using analysis kits from Helena Laboratories (USA). Serum catalase was determined spectrophotometrycally by the method of Aebi [39]. Serum superoxide dismutase (SOD) was determined spectophotometrically by a coupled assay using a kit supplied by Cayman Chemical Company (USA). Lipid peroxidation was assessed by reaction of malondialdehyde (MDA) with thiobarbituric acid according to the method of Ohkawa *et al*. [40]. Total serum and tissue protein levels were determined by the Coomassie Blue dye binding method of Bradford [41].

### Statistical analysis

Data are expressed as mean +/− SEM. Statistical analysis was carried out using SPSS V. 21 (IBM). Readings within each group were compared using the independent sample test, whereas the readings of the different groups were compared using One-Way ANOVA: Post hoc multiple comparisons test. In Post Hoc, equal variances were assumed by Fisher’s Least Significant Difference, (LSD) with *P* < *0.05* being significant.

## Results

### Effect of AGE on physical measurements

**Fig. 1:**
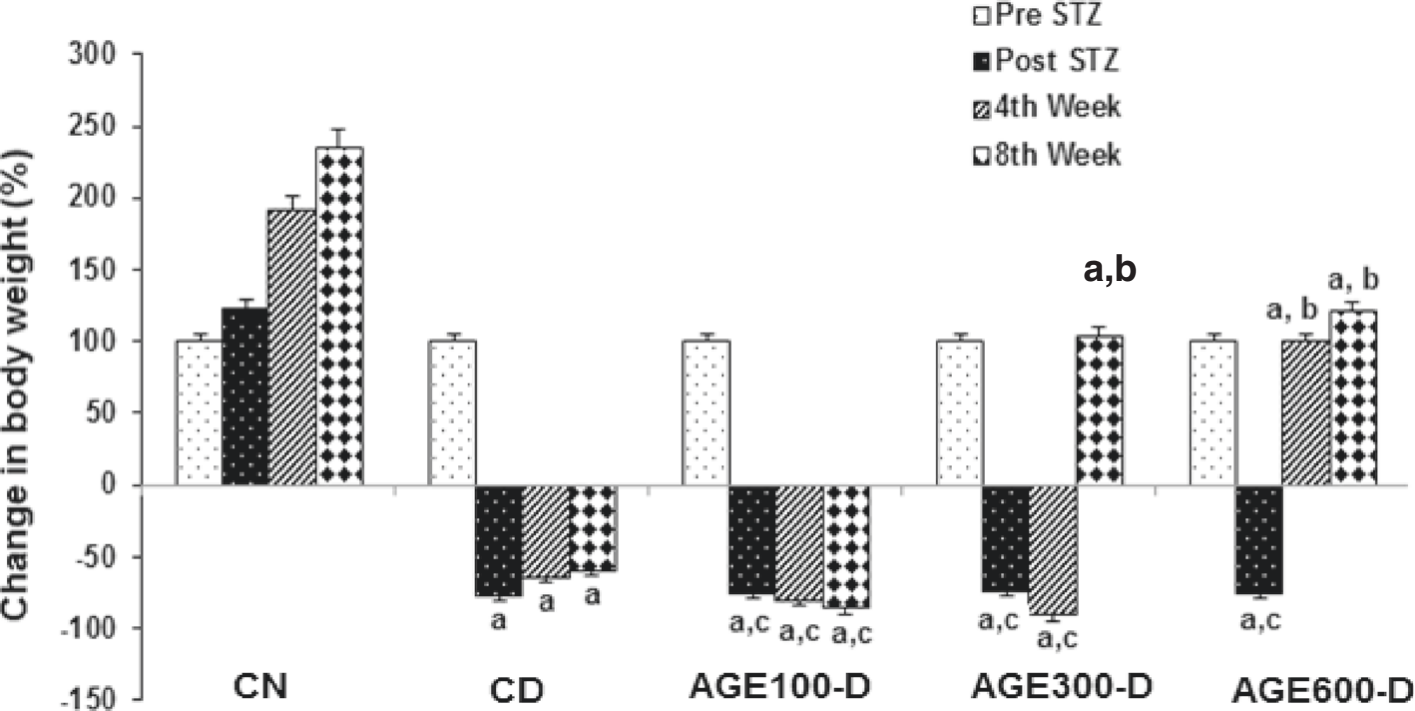
Dose response of aged garlic extract (AGE) on body weight in diabetic rats. AGE at doses of 100 mg/kg, 300 mg/kg and 600 mg/kg were administered IP to diabetic rats for 8 weeks abbreviated as AGE100-D, AGE300-D and AGE600-D, respectively. Diabetic control rats (CD) and normal control rats (CN) were administered normal saline. The animals were weighed before STZ injection (Pre STZ), 1 week after STZ injection (Post STZ) and at weeks 4 and 8 of the experiment. Weights are plotted as percentiles with the starting weights all standardized to 100 %. *a* = significantly decreased compared to normal saline rats; *b* = significantly increased compared to diabetic control; *c* = not significantly different from diabetic control

During the 8 weeks of the study, the CN rats more than doubled in weight (Fig. 1) while the CD rats lost weight and were over 50 % lighter at 8 weeks compared to the start of the experiment. In comparison, AGE-D rats recovered in terms of body weight after an initial loss following STZ injection in a dose–dependent manner with a significant increase in weight in AGE300-D and AGE600-D treatment groups compared to CD rats.

The CD rats greatly increased water (235 ± 3 ml/24 h) and food (46 ± 2 g/24 h) intake during the experiment compared to CN rats (40 ± 1 ml/24 h, 30 ± 1 gm/24, respectively) (Table 1). AGE treatment of diabetic rats at a dose of 600 mg/kg led to a significantly less food (34 ± 3 gm/24 h) and water (185 ± 2 ml/24 h) intake in comparison to CD rats. The two lower doses of AGE did not have an effect on either food or water intake.

**Table 1:** Dose response of aged garlic extract (AGE) on 24 h urine output, food intake and water intake in diabetic rats

Group	n	Urine output (ml/24 h)	Food intake (gm/24 h)	Water intake (ml/24 h)
CN	12	10 ± 1	30 ± 1	40 ± 1
CD	12	95 ± 2^a^	46 ± 2^a^	235 ± 3^a^
AGE 600-D	16	87 ± 2^a,b^	34 ± 3^a,b^	185 ± 2^a,b^
AGE 300-D	15	89 ± 3^a,b^	41 ± 2^a,c^	225 ± 1^a,c^
AGE 100-D	15	92 ± 1^a, c^	41 ± 3^a,c^	232 ± 1^a,c^

a = significantly decreased compared to normal saline rats; b = significantly increased compared todiabetic control; c = not significantly different from diabetic control

In comparison, urine output (Table 1) in CD rats was over 9 times (95 ± 2 ml/24 hrs) the output in CN animals (10 ± 1 ml/24 hrs). The diabetic animals treated with AGE at 300 and 600 mg/kg had significantly reduced urine output (89 ± 3 and 87 ± 2 ml/24 hrs, respectively). In comparison, the lowest dose of AGE (100 mg/kg) did not have an effect on urine output (92 ± 1 ml/24 hrs) compared to CD rats.

### Effect of AGE on blood glucose, glycated hemoglobin and serum insulin

**Fig. 2:**
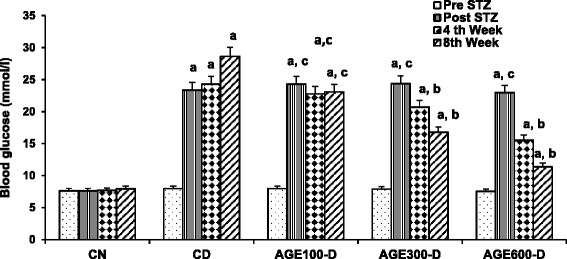
Dose response of aged garlic extract (AGE) on blood glucose levels in diabetic rats. AGE at doses of 100 mg/kg, 300 mg/kg and 600 mg/kg were administered IP to diabetic rats for 8 weeks abbreviated as AGE100-D, AGE300-D and AGE600-D, respectively. Diabetic control rats (CD) and normal control rats (CN) were administered normal saline. Blood glucose levels were measured before STZ injection (Pre STZ), 1 week after STZ injection (Post STZ) and at weeks 4 and 8 of the experiment. *a* = significantly increased compared to normal saline rats; *b* = significantly decreased compared to diabetic control; *c* = not significantly different from diabetic control

As shown in Fig. 2, blood glucose levels in untreated CD rats continued to increase throughout the experimental period, while glucose levels in diabetic rats treated with 300 or 600 mg/kg of AGE decreased significantly. In contrast, blood glucose levels in diabetic rats treated with 100 mg/g dose of AGE were not significantly different from the readings of the CD rats.

**Fig. 3:**
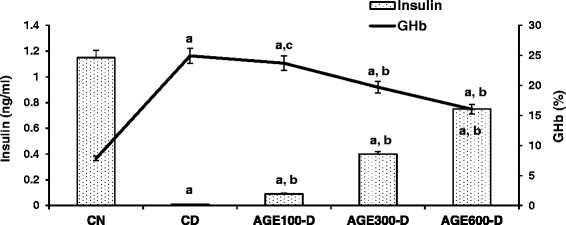
Dose response of aged garlic extract (AGE) on serum insulin and RBC GHb in diabetic rats. AGE at doses of 100 mg/kg, 300 mg/kg and 600 mg/kg were administered IP to diabetic rats for 8 weeks abbreviated as AGE100-D, AGE300-D and AGE600-D, respectively. Diabetic control rats (CD) and normal control rats (CN) were administered normal saline. Serum insulin levels and glycated hemoglobin (GHb) in red blood cells were measured at the conclusion of the treatment period (8 weeks). *a* = significantly different compared to normal saline rats; *b* = significantly different compared to diabetic control; *c* = not significantly different from diabetic control

As shown in Fig. 3, induction of diabetes with STZ resulted in approximately 11-fold reduction in serum insulin level (0.01 ± 0.00 ng/ml) compared to NC rats (1.15 ± 0.02 ng/ml). AGE treatment of diabetic rats with doses of 100, 300 or 600 mg/kg resulted in a higher serum insulin with increases of 9 % (0.09 ± 0.01 ng/ml), 34 % (0.40 ± 0.03 ng/ml) and 64 % (0.75 ± 0.05 ng/ml), respectively. In contrast and in agreement with serum glucose levels, GHb was significantly higher in CD rats (24.9 ± 1.7 %) compared to CN rats (7.8 ± 0.4 %). GHb levels were significantly decreased by the 300 and 600 mg/kg doses of AGE (19.7 ± 0.8 and 16.0 ± 1.2, respectively), while the 100 mg/kg dose of AGE did not have an effect on this parameter.

### Effect of AGE on serum triglyceride and total cholesterol

**Fig. 4:**
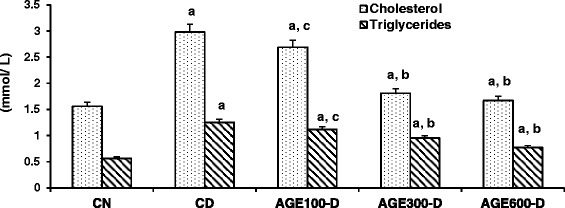
Dose response of aged garlic extract (AGE) on serum total cholesterol and triglycerides in diabetic rats. AGE at doses of 100 mg/kg, 300 mg/kg and 600 mg/kg were administered IP to diabetic rats for 8 weeks abbreviated as AGE100-D, AGE300-D and AGE600-D, respectively. Diabetic control rats (CD) and normal control rats (CN) were administered normal saline. Serum total cholesterol and triglyceride levels were measured at the conclusion of the treatment period (8 weeks). *a* = significantly increased compared to normal saline rats; *b* = significantly decreased compared to diabetic control; *c* = not significantly different from diabetic control

Figure 4 shows that serum total cholesterol (3.0 ± 0.1 mM) and triglycerides (1.25 ± 0.10 mM) were significantly increased in CD rats compared to CN rats (1.6 ± 0.04 and 0.57 ± 0.03 mM, respectively). The 2 higher doses of AGE significantly lessened the increase in the level of cholesterol (1.7 ± 0.1 and 1.8 ± 0.1 mM, respectively) and triglycerides (0.77 ± 0.07 and 0.95 ± 0.09 mM, respectively) compared to CD, while treatment of diabetic rats with the 100 mg/kg dose was not effective in attenuating the increases in either cholesterol (2.7 ± 0.08 mM) or triglycerides (1.12 ± 0.03 mM) in serum.

### Effect of AGE on oxidative stress in serum, kidney and liver

**Fig. 5:**
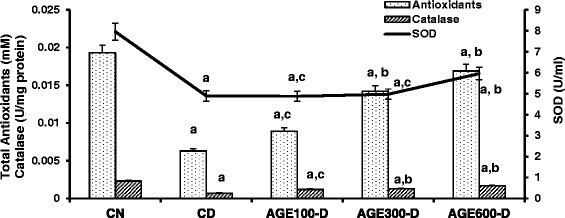
Dose response of aged garlic extract (AGE) on serum total antioxidants, catalase and SOD in diabetic rats. AGE at doses of 100 mg/kg, 300 mg/kg and 600 mg/kg were administered IP to diabetic rats for 8 weeks abbreviated as AGE100-D, AGE300-D and AGE600-D, respectively. Diabetic control rats (CD) and normal control rats (CN) were administered normal saline. Serum total antioxidants (mM), catalase activity (U/mg protein) and SOD activity (U/ml) were determined at the conclusion of the treatment period (8 weeks). *a* = significantly decreased compared to normal saline rats; *b* = significantly increased compared to diabetic control; *c* = not significantly different from diabetic control

Figure 5 illustrates that serum total anti-oxidants, catalase and superoxide dismutase activities (SOD) were significantly decreased in CD rats (0.0063 ± 0.0005 mM, 0.0007 ± 2 X 10^−6^ U/mg protein, 4.89 ± 0.34 U/ml, respectively) compared to CN rats (0.02 ± 0.01 mM, 0.0023 ± 4 X 10^−5^ U/mg protein, 7.95 ± 0.62 U/ml, respectively). Total serum antioxidants and catalase activity were significantly increased by treatment of diabetic rats with 300 (0.14 ± 0.009 mM, 0.0013 ± 3 x 10^−5^ U/mg protein, 4.97 ± 0.33 U/ml) and 600 mg/kg of AGE (0.017 ± 0.01 mM, 0.002 ± 4 X 10^−5^ U/mg protein, respectively). Serum SOD activity was only significantly increased in diabetic rats treated with 600 mg AGE/kg. The lowest AGE dose (100 mg/kg) had no effect on serum antioxidant parameters.

**Fig. 6:**
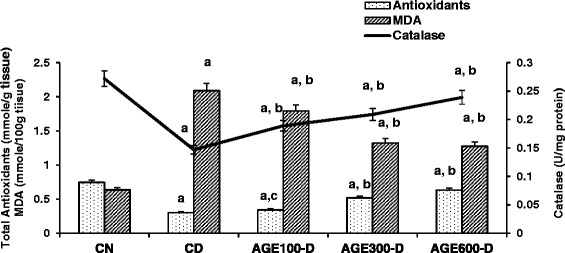
Dose response of aged garlic extract (AGE) on kidney total antioxidants, catalase and MDA in diabetic rats. AGE at doses of 100 mg/kg, 300 mg/kg and 600 mg/kg were administered IP to diabetic rats for 8 weeks abbreviated as AGE100-D, AGE300-D and AGE600-D, respectively. Diabetic control rats (CD) and normal control rats (CN) were administered normal saline. Total antioxidants (mmole/g tissue), catalase activity (U/mg protein) and MDA levels (mmole/100 g tissue) were determined in kidney tissue at the conclusion of the treatment period (8 weeks). *a* = significantly diferent compared to normal saline rats; *b* = significantly different compared to diabetic control; *c* = not significantly different from diabetic control

**Fig. 7:**
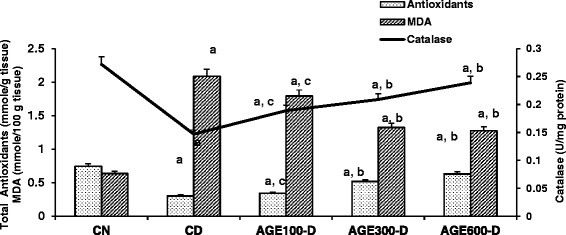
Dose response of aged garlic extract (AGE) on liver total antioxidants, catalase and MDA in diabetic rats. AGE at doses of 100 mg/kg, 300 mg/kg and 600 mg/kg were administered IP to diabetic rats for 8 weeks abbreviated as AGE100-D, AGE300-D and AGE600-D, respectively. Diabetic control rats (CD) and normal control rats (CN) were administered normal saline. Total antioxidants (mmole/g tissue), catalase activity (U/mg protein) and MDA levels (mmole/100 g tissue) were determined in liver tissue at the conclusion of the treatment period (8 weeks). *a* = significantly different compared to normal saline rats; *b* = significantly different compared to diabetic control; *c* = not significantly different from diabetic control

In parallel with serum anti-oxidants and catalase, total kidney and liver anti-oxidants and catalase activity were significantly lower in CD rats (Figs. 6 and 7). Treatment with AGE at doses of 300 and 600 mg/kg resulted in significantly higher kidney and liver anti-oxidants and catalase activity. In contrast, kidney and liver MDA levels were significantly higher in CD rats compared to CN rats. AGE-treated rats with the 300 and 600 mg/kg doses exhibited significantly decreased kidney and liver MDA levels. Note that the lowest dose of AGE (100 mg/kg) significantly increased kidney catalase activity and decreased kidney MDA levels, but had no effect on these anti-oxidant markers in liver tissues.

## Discussion

During the last two decades, considerable research has been devoted to studying the effects of natural herbs and spices as alternative forms of treatment for chronic diseases such as DM. Extensive research has established that fresh garlic extract and some garlic constituents have anti-diabetic and anti-oxidant properties. However, AGE, an over the counter available stable garlic preparation with known composition, in addition to acceptable ingestion and side effects attributes, has received little attention as an anti-diabetic agent. The goal of the present study was to assess the anti-diabetic and related anti-oxidant potential of AGE, which is either lacking in certain aspects or controversially reported in with respect to other aspects in the literature. Toward that objective and to define the most active dose or range of doses, a three incremental-dose–response study of AGE was investigated in STZ-induced diabetic rats.

Prolonged hyperglycemia in both human conditions and animal models has been shown to result in the physical symptoms of diabetes including weight loss, polyphagia, polydipsia and polyuria [42, 43]. As expected, in the present study, the STZ-induced CD rats rapidly developed and maintained these physical symptoms and exhibited over 50 % weight loss during the 8 week experiment. The lowest dose of AGE (100 mg/kg) did not have a marked effect on these parameters, while the AGE-D rats treated with the 300 or 600 mg/kg doses stabilized their weight after an initial weight loss, in addition to an improved polydipsia, polyphagia and polyuria in diabetic rats treated with 600 mg AGE/kg. Lessening of polyuria in response to AGE is consistent with the results of Shiju and coworkers [36] who reported amelioration of polyuria in a 500 mg/kg oral AGE treated STZ-induced diabetic rats.

In the present study, STZ-injected rats developed a rapid hyperglycemia and hypoinsulinemia as shown previously by our group and others [44–46]. Treating diabetic rats with either 300 or 600 mg/kg dose of AGE significantly decreased blood glucose and markedly increased serum insulin. These results are in contrast with those of Shiju *et al*. [36] who did not observe any hypoglycemic effect of AGE. However, with respect to the anti-glycation effects, both the current study and that of Shiju *et al.* [36] showed that AGE treatment leads to a decrease in GHb. Thus, these results suggest that AGE may prevent formation of Amadori products which can lead to protein oxidation. This conclusion is supported by reports that AGE has potent anti-glycation potential, which may be one of the mechanisms by which AGE exerts its protective action in DM [47, 48].

Dyslipidemia, a major symptom and risk factor for the development of cardiovascular diseases in DM [49, 50] is characterized by high serum levels of both triglycerides and cholesterol. Previous studies in both humans and animals have reported that AGE has a lipid-lowering property [51]. Similarly, in the present study and as reported by Shiju *et al*. [36], treatment with all doses of AGE led to a significant attenuation of the elevation of both serum triglycerides and total cholesterol observed in the CD rats. Interestingly, treatment of type 2 diabetic patients with 3000 mg of AGE daily had no effect on serum cholesterol but did slightly, although not significantly, lower serum triglycerides after 3 months of treatment [37]. These observations in the type-2 diabetes suggest that part of the AGE ameliorative mechanism of action on body lipids may be mediated at the cellular level.

Oxidative stress has been implicated as a key contributor to many diabetic complications, including nephropathy [26]. Thus, alleviation of OS is expected to reverse or at least lessen many of the negative impacts of DM. A number of studies have investigated the anti-oxidant potential of AGE and have suggested that AGE attenuates OS by increasing endogenous anti-oxidant enzymes, such as catalase and glutathione peroxidase [22]. In addition, *in vitro* and *in vivo* studies have indicated that AGE can reduce both the formation of superoxide and lipid peroxidation, and thus alleviate OS [24]. In spite of the established connection between OS and DM, the anti-oxidant potential of AGE has not previously been studied in DM. In the current study, indicators of OS were assessed in serum as well as kidney and liver tissues of STZ-induced diabetic rats. As we have previously reported, anti-oxidants were decreased and OS was increased in serum, liver and kidneys of this experimental model of CD rats [31, 44]. Treatment with either 300 or 600 mg/kg of AGE significantly increased the anti-oxidant defense systems as indicated by increased levels of total anti-oxidants and anti-oxidant enzymes and lowered lipid peroxidation in liver and kidney tissues. In agreement, Balamash *et al.* [37] also reported decreased lipid peroxidation in AGE-treated diabetic patients.

In an attempt to specify the active components of AGE, Saravanan *et al*. [50] investigated the anti-diabetic properties of SAC, the major garlic-derived component found in AGE, in STZ-induced diabetic rats. Those investigators reported that SAC at 100 and 150 mg/kg decreased blood glucose and GHb and increased serum insulin. Thus, Saravanan and coworkers [52] suggested that SAC may be responsible for the anti-diabetic effect of AGE. The increase in insulin concentration induced by AGE may be the mechanism by which SAC brings about its actions. This mechanism of action may not be different from that suggested for fresh garlic. Therefore, we propose that the anti-diabetic actions of AGE are due to amendment of the insulin-glucose biochemical pathway. This point of view is based on the fact that many diabetic symptoms are consequences of cellular abnormalities resulting from hypoinsulinemia-hyperglycemia. The different doses of AGE tested gave some indication as to the potency of this natural product preparation, in addition to suggesting a suitable and tolerable dose that can be taken as a supplement by diabetics. Although AGE has many of the same active components as fresh garlic, the process of aging may have led to attenuation in the potency of these active components. For that reason, a larger dose of AGE (600 mg/kg) may be required to induce almost similar actions to those induced by a slightly lower dose of fresh garlic (500 mg/kg).

## Conclusion

In conclusion, the observed attenuating effects of AGE on the diabetic indicators in STZ-induced diabetic rats suggest that this ready-to-use, fixed concentration, stable preparation has the potential to be used as a palatable anti-diabetic agent. Further biochemical and pharmacological investigations into the insulin-glucose pathway are necessary to further elucidate the anti-diabetic and connected anti-oxidant mechanism of action of AGE to support its use as a possible supplementary treatment in DM.

## Abbreviations

AGE: Aged garlic extract
AGE100-D: Low dose AGE-diabetic
AGE300-D: Medium dose AGE-diabetic
AGE600-D: High dose AGE-diabetic
CD: Control diabetic
CN: Control normal
DM: Diabetes mellitus
GHb: Glycated hemoglobin
GPx: Glutathione peroxidase
NS: Normal saline
OS: Oxidative stress
SAC: S-allylcysteine
SAMC: S-allylmercaptocysteine
SOD: Superoxide dismutase
STZ: Streptozotocin
